# Colonization with Altered Schaedler Flora Impacts Leukocyte Adhesion in Mesenteric Ischemia-Reperfusion Injury

**DOI:** 10.3390/microorganisms9081601

**Published:** 2021-07-27

**Authors:** Franziska Bayer, Stefanie Ascher, Klytaimnistra Kiouptsi, Jens M. Kittner, Roland H. Stauber, Christoph Reinhardt

**Affiliations:** 1 Center for Thrombosis and Hemostasis (CTH), University Medical Center Mainz, Johannes Gutenberg-University Mainz, Langenbeckstrasse 1, 55131 Mainz, Germany; franziska.bayer@uni-mainz.de (F.B.); stefanie.kemski@hotmail.de (S.A.); klytaimnistra.kiouptsi@unimedizin-mainz.de (K.K.); 2 Department of Chemistry, Biochemistry, Johannes Gutenberg-University Mainz, 55128 Mainz, Germany; 3 Department of Medicine, University Medical Center Mainz, Johannes Gutenberg-University Mainz, Langenbeckstrasse 1, 55131 Mainz, Germany; jenskittner@yahoo.de; 4 Diakonie Klinikum Neunkirchen, Brunnenstraße 20, 66538 Neunkirchen, Germany; 5 Department of Nanobiomedicine/ENT, University Medical Center Mainz, Johannes Gutenberg-University Mainz, Langenbeckstrasse 1, 55131 Mainz, Germany; rstauber@uni-mainz.de; 6 German Center for Cardiovascular Research (DZHK), Partner Site RheinMain, 55131 Mainz, Germany

**Keywords:** microbiota, germ-free, altered Schaedler flora, acute mesenteric infarction, mesenteric ischemia-reperfusion injury, leukocytes, thrombosis, C–C motif chemokine ligand 5

## Abstract

The microbiota impacts mesenteric ischemia-reperfusion injury, aggravating the interaction of leukocytes with endothelial cells in mesenteric venules. The role of defined gut microbiomes in this life-threatening pathology is unknown. To investigate how a defined model microbiome affects the adhesion of leukocytes in mesenteric ischemia-reperfusion, we took advantage of gnotobiotic isolator technology and transferred altered Schaedler flora (ASF) from C3H/HeNTac to germ-free C57BL/6J mice. We were able to detect all eight bacterial taxa of ASF in fecal samples of colonized C57BL/6J mice by PCR. Applying qRT-PCR for quantification of species-specific 16S rDNA sequences of ASF bacteria, we found a major shift in the abundance of ASF 500, which was greater in C57BL/6J mice relative to the C3H/HeNTac founder breeding pair. Using high-speed epifluorescence intravital microscopy to visualize the venules of the small bowel mesentery, we found that gnotobiotic ASF-colonized mice showed reduced leukocyte adherence, both pre- and post-ischemia. Relative to germ-free mice, the counts of adhering leukocytes were increased pre-ischemia but did not significantly increase in ASF-colonized mice in the post-ischemic state. Collectively, our results suggest a protective role of the minimal microbial consortium ASF in mesenteric ischemia-reperfusion injury.

## 1. Introduction

The commensal gut microbiota is a complex microbial ecosystem, established at birth, and developing a mutualistic relationship with its host [1,2]. The investigation of physiological microbiota–host interactions is largely hampered by the sheer complexity of this ecosystem, consisting of 400–1000 microbial species that depend on the presence of one another, e.g., due to microbial cross-feeding [3]. Hence, the investigation of the causal impact of individual gut microbes on host physiology requires experimentation with germ-free (GF) mouse models, in particular as antibiotic depletion of the gut bacteria is insufficient to specifically pinpoint microbiota–host interactions [4,5]. Therefore, gnotobiotic approaches with model microbiomes have become an essential element to clarify how defined microbiota or individual microbial species impact host physiology [6].

In recent years, several model microbiomes with well-defined composition have been developed that closely mimic the mouse or human intestinal microbiome. This approach is particularly useful to avoid influences by uncharacterized microbes or to overcome the lack of reference strains. For example, the mouse intestinal bacterial collection (miBC) contains 100 strains, representing 76 different species form 26 families, belonging to the phyla Actinobacteria, Baceroidetes, Firmicutes, Proteobacteria, and Verrucomicrobia [7]. Although fairly comparable to the mouse or human intestinal microbiota, a main disadvantage remains the enormous complexity of such a model microbiota. For this reason, even if considered artificial, simple microbial consortia, such as the altered Schaedler flora (ASF) have the decisive advantage of reduced complexity, making them suitable for functional microbiome studies [8]. ASF consists of ASF 356 (*Clostridium* sp.), ASF 360 (*Lactobacillus intestinalis*), ASF 361 (*Lactobacillus murinus*), ASF 457 (*Mucispirillum schaedleri*), ASF 492 (*Eubacterium plexicaudatum)*, ASF 500 (*Pseudoflavinifractor* sp.), ASF 502 (*Clostridium* sp.), and ASF 519 (*Parabacteroides goldsteinii*) [9,10]. Furthermore, specific native model microbiomes, containing only a few bacterial species, been have developed, e.g., SIHUMI with seven species of the human intestine or MIBAC-1 with 18 species of the mouse intestine [7,11]. In the case of ASF, all eight bacterial species are well-characterized and can be cultured, and this model microbiome has proven stable over decades [10]. While colonization with ASF represents a reductionist approach, due to its known functions, it certainly constitutes a valid model to understand how distinct gut commensals can influence host (patho)physiology.

The gut microbiota serves a multitude of vital functions, such as the harvest of otherwise inaccessible nutrients, the control of renewal processes of the intestinal epithelium, and immune education [12,13,14]. Since the gut microbiota coevolves with the host, changes in diet and lifestyle are an important evolutionary selection pressure on the gut microbiome. Recently, exposure to naturally occurring or intentionally added nanoparticles were recognized as a factor that may modify the composition and diversity of the commensal microbiome, and therefore also affect human health [15,16,17,18]. At present, the complex interaction of nanoparticles with microbes and the potential beneficial or detrimental (patho)biological consequences remain to be explored [19].

In addition to the beneficial traits on host physiology, the gut microbiota can also contribute to the development of metabolic and cardiovascular disease phenotypes, as well as allergies [20,21,22,23,24]. The gut microbiota was demonstrated to influence vascular physiology on multiple levels. Colonization with a gut microbiota triggers vascular remodeling and the formation of intricate capillary networks and lacteals in the small intestinal mucosa [25,26,27]. The expression of intercellular adhesion molecule-1 (ICAM-1) in the splanchnic circulation, a major determinant of leukocyte-endothelial cell interactions, is constitutively upregulated by the presence of gut commensals [28]. The microbiota affects vascular inflammation and the development of atherosclerotic lesions, both through its influence on immune cell-mediated mechanisms but also by its impact on lipid metabolism [29,30,31,32,33]. Moreover, the microbiota promotes arterial thrombus growth through various mechanisms [34,35,36]. Therefore, the microbiota needs to be considered an environmental factor influencing vascular pathology.

The commensal microbiota is increasingly recognized as a modifier of ischemic disease states [37,38,39]. Acute mesenteric infarction is characterized by a disrupted gut barrier and dysregulation of the host immune response [40,41]. It requires immediate treatment to prevent life-threatening complications, such as peritonitis and multiple organ failure [42,43]. In recent work with gnotobiotic mouse models, we revealed that the presence of gut microbiota enhances leukocyte adhesion to ischemia-reperfusion injured mesenteric venules, but efficiently suppresses the formation of neutrophil extracellular traps (NETs) [44]. This was regulated through toll-like receptor-4 (TLR4) signaling. In line, previous work with GF mice has demonstrated a protective role of microbiota-triggered nucleotide-binding oligomerization domain-containing protein 2 (NOD2) signaling in ischemia-reperfusion-induced intestinal injury [45]. Furthermore, the probiotic formulation VSL#3, mediated via myeloid differentiation primary response 88 (MyD88)-mediated signaling, delayed thrombus formation in cremaster muscle arterioles at conditions of acute intestinal inflammation [46]. While the pro-adhesive role of a complex gut microbiota for leukocyte tethering in ischemia-reperfusion injury is well-recognized [44,47], the protective function of defined gut bacterial strains remains unresolved.

Allowing for the development of a relatively normal immune system and gastrointestinal function [48,49], we transferred the minimal microbial consortium ASF from C3H mice onto GF C57BL/6J mice. We aimed to test the impact of the defined minimal microbial consortium ASF on leukocyte adhesion in mesenteric ischemia-reperfusion injury; we imaged mesenteric venules by high-speed intravital microscopy. Ischemia-reperfusion injury-induced leukocyte adhesion to the endothelium of mesenteric venules was abolished in ASF-colonized mice compared to GF or conventionally raised (CONV-R) controls.

## 2. Materials and Methods

*Animals.* The C57BL/6J and C3H/HeNTac mouse strains were used for the experiments. Germ-free (GF) C57BL/6J mice, GF C57BL/6J mice colonized with altered Schaedler flora (ASF), and ASF harboring C3H/HeNTac mice were maintained as a gnotobiotic mouse colony in sterile flexible film isolators. The germ-free state was verified every second week by PCR for detection of 16S rDNA and by bacterial culture. Conventionally raised (CONV-R) C57BL/6J mice were sourced from the same colony and kept under specific-pathogen-free (SPF) conditions. In order to transfer the ASF, three male and female GF C57BL/6J mice aged 13 to 16 weeks were colonized with fecal contents harvested from one female and one male C3H/HeNTac mouse, and were dissolved in 5 mL of sterile PBS and 250 µL gavaged once. For intravital microscopy experiments and expression analyses, all experimental animals were between 5–12 weeks of age. Mice were housed in the Translational Animal Research Center (TARC) of the University Medical Center Mainz under SPF conventionally raised (CONV-R) or germ-free (GF) housing conditions in EU type II cages, with 2–5 cage companions with a standard autoclaved lab diet and water ad libitum, 22 ± 2 °C room temperature and a 12 h light/dark cycle. All groups of mice were sex and age-matched and were free of clinical symptoms. All procedures on mice were approved by the local committee on legislation of animals (Landesuntersuchungsamt Rheinland-Pfalz, Koblenz, Germany; G11-1-025, G13-1-035, G13-1-072, and G16-1-013).

*Collection of fecal samples and isolation of genomic DNA.* Two fresh fecal pellets were collected in one sterile Eppendorf tube directly from the animals and stored until further usage at −20 °C. At least two micro tubes with feces were collected on each sampling day from each mouse. Genomic DNA was isolated using a NucleoSpin Soil Kit (Macherey-Nagel, Düren, Germany), according to the protocol and stored at −20 °C.

*PCR and qPCR analysis for detection of bacterial taxa.* For PCR analysis, DreamTaq Hot Start Green MasterMix (ThermoFisher Scientific, Waltham, MA, USA) and primer pairs published by Gomes-Neto with a final concentration of 300 nM were used. Reaction conditions included: an initial denaturation step of 4 min at 95 °C, followed by 40 cycles of 30 s at 95 °C, 30 s at 58 °C and 20 s at 72 °C, and one cycle of 5 min at 72 °C, using a Mastercycler Pro S PCR Thermal Cycler (Eppendorf, Hamburg, Germany). Each reaction contained 23 µL PCR mix and 2 µL gDNA (10 ng). Optimal annealing temperatures and primer concentrations were based on the results of gradient PCR reactions. For qPCR analysis, iTaq Universal SYBR Green Supermix (BioRad, Hercules, CA, USA) and primer pairs with a final concentration of 300 nM were used [50] (Table 1). Reaction conditions included: initial denaturation step of 3 min at 95 °C, followed by 40 cycles of 15 s at 95 °C and 20 s at 58 °C, followed by a melting curve, using qTOWER3 G (Analytic Jena, Jena, Germany). Each reaction contained 16 µL qPCR mastermix and 4 µL gDNA (20 ng).

*Isolation of intestinal epithelial cells (IECs).* The last third of the small intestine was removed and flushed with ice-cold PBS. Until further processing, small intestine segments were stored in 15 mL Falcons with 5 mL PBS on ice. Segments were cut open lengthwise and washed once in PBS. Washed segments were put in 4.5 mL pre-warmed 10 mM EDTA/PBS solution and incubated for 30 min at 37 °C. Vigorous shaking by hand or vortex produced a white-opaque solution, which was then divided into two 2 mL reaction vials. After a centrifugation step of 7500 rpm for 7 min at 4 °C, the supernatant was decanted and cells were resuspended with 1 mL ice-cold PBS. The cell suspension was centrifuged at 7500 rpm for 7 min at 4 °C. 350 µL RIPA-buffer including protease- and phosphatase inhibitors were added, the samples were resuspended and shock frozen in liquid nitrogen.

*Protein extraction.* Samples were thawed on ice, resuspended, and incubated for 15 min on ice. The samples were sonicated for 1 min, incubated again for 15 min on ice, and sonicated again for 1 min. The samples were centrifuged at 10.000× *g* for 15 min at 4 °C. The supernatant was pipetted into a new vial. The total protein concentration was determined with the DC protein Assay (BioRad, USA), following the assay protocol. For CCL5 ELISA, all samples had a protein concentration of 80 µg in 200 µL.

*ELISA.* CCL5 ELISA was performed according to the manufacturer’s instructions (Abcam, ab100739).

*RNA isolation of small intestine.* Samples were thawed on ice, two 7 mm stainless steel beads (Qiagen, Hilden, Germany, #1057465) and 1 ml Tri-reagent (Thermo Fisher Scientific, USA) were added. Tissues were disrupted using a TissueLyser II machine (Qiagen, Germany) using pre-cooled plates (−20 °C) with the following times: 2 min at 30 Hz, 1 min cool down at −80 °C, 2 min at 30 Hz. After samples were disrupted, they were incubated at room temperature for 5 min. Then, 0.2 mL chloroform was pipetted to each sample, vortexed for 15 s, and incubated for 3 min. To separate the phases, samples were centrifuged at 12,000× *g* at 4 °C for 15 min. The supernatant was transferred into a new vial and 0.6 mL isopropanol was added. Then it was mixed and incubated for 10 min. The mixture was centrifuged at 12,000× *g* at 4 °C for 10 min, the supernatant decanted, and 1 mL of 75% ethanol was added. The samples were vortexed for 5 s and centrifuged at 7500× *g* at 4 °C for 5 min. The supernatant was decanted again, 1 mL of 75% ethanol was added, vortexed for 5 s, and centrifuged at 7500× *g* at 4 °C for 5 min. Once more the supernatant was decanted and the pellet was left to dry for 7 min, before adding 30 µL diethylpyrocarbonate (DEPC)-water. RNA integrity was analyzed by denaturing agarose gels and Nanodrop (Thermo Fisher Scientific).

*qRT-PCR for CCL5.* Samples were diluted to 200 ng/µL RNA and cDNA was transcribed using a high capacity cDNA kit by Applied Biosystems, according to the manufacturer’s protocol. cDNA was diluted to 5 ng/µL with RO-H_2_O. qPCR was performed as described before, using the following primers: mouse *CCL5* forward GTGCTCCAATCTTGCAGTCG and mouse *CCL5* reverse AGAGCAAGCAATGACAGGGA.

*Mesenteric ischemia-reperfusion injury model*. Citrate whole blood was collected by intracardial puncture. To characterize leukocyte vessel wall interactions in vivo, acridine orange (50 µg/µL, 50 µL per mouse, Sigma-Aldrich St. Louis, MO, USA) stained leukocytes were imaged [42]. Mice were anesthetized by i.p. injection of a mixture of midazolame (5 mg/kg, hameln pharma plus, Hameln, Germany), medetomidin (0.5 mg/kg, Zoetis, Parsippany, NJ, USA), and fentanyl (0.5 mg/kg, Janssen-Cilag GmbH, Neuss, Germany). A polyethylene catheter (0.28 mm ID, 0.61 mm OD, Smiths Medical Deutschland GmbH, Grasbrunn, Germany) was implanted into the jugular vein. The abdomen of CONV-R mice, GF mice, and descendant gnotobiotic mice of the established ASF colony was entered via a midline laparotomy incision. The superior mesenteric artery was identified and occluded with a small vascular clamp. After an ischemic interval of 60 min, reperfusion was allowed. Before and immediately after ischemia-reperfusion, the entire small intestine was carefully taken out of the abdomen. Leukocytes were visualized in situ by in vivo epifluorescence high-speed video microscopy in the mesenterial venules. Animals were sacrificed by cervical dislocation at the end of the experiments. All procedures performed on mice were approved by the local committee for legislation on the protection of animals (Landesuntersuchungsamt Rheinland-Pfalz, Koblenz, Germany; G13-1-072 and G16-1-013).

*Intravital high-speed video epifluorescence microscopy.* Intravital microscopy was performed using a high-speed wide-field Olympus BX51WI fluorescence microscope with a long-distance condenser and a 10× (NA 0.3, BG filter) water immersion objective with a monochromator (MT 20E, Olympus Deutschland GmbH, Hamburg, Germany) and a charge-coupled device camera (ORCA-R2, Hamamatsu Photonics, Hamamatsu City, Japan). For image acquisition and analysis, the Real-time Imaging System eXcellence RT (Olympus Deutschland GmbH, Hamburg, Germany) software was used. Leukocyte adherence was quantified in one field view of 0.06 mm^2^. Adherent leukocytes were defined as cells that did not move or detach from the endothelial lining within an observation period of 20 s.

*Statistical analysis.* Data are presented as mean ± S.E.M and were analyzed with GraphPad Prism 8 (GraphPad Software Inc., San Diego, CA, USA). The D’Agostino-Pearson omnibus K-squared test was performed to determine the normality of the data and the F test was used to determine the equality of variances. The independent samples Student’s *t*-test was applied for comparison of two groups. One-way ANOVA was applied for comparisons of more than two groups. If data were not normally distributed, a nonparametric Mann–Whitney test was used.

## 3. Results

### 3.1. Successful Transfer of Altered Schaedler Flora from C3H/HeN Mice on the C57BL/6J Background, with Mouse Strain-Specific Impact on the Abundance of ASF 500

**Figure 1:**
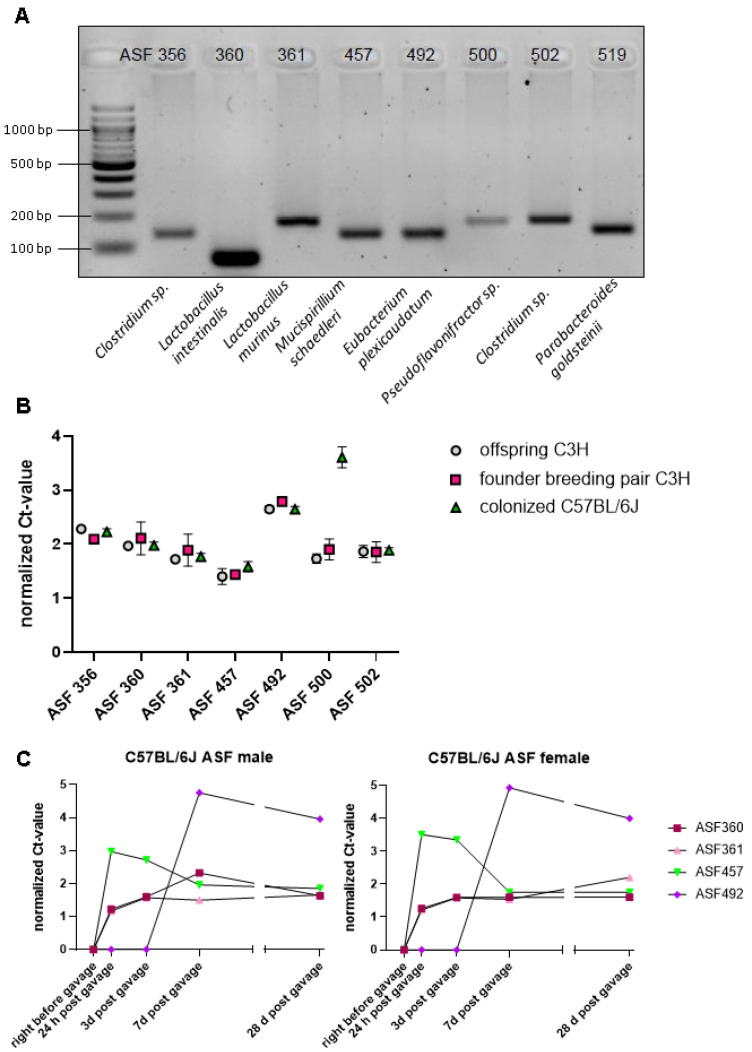
Transfer of ASF from C3H/HeNTac mice onto C57BL/6J mice. (**A**). PCR based detection of ASF bacteria, performed with published primer pairs [50]. All ASF-specific bacteria were detected. (**B**). qRT-PCR based detection of ASF bacteria, performed with published primer pairs [50]. The graph compares the founder breeding pair (C3H/HeNTac; aged 20 weeks; pink, N = 2) with the C3H/HeNTac ASF offspring (aged 4 weeks; grey, N = 4) and the newly colonized C57BL6J mice (aged 17–19 weeks; green, N = 2). (**C**). Three male and female GF C57BL/6J mice aged 13–16 weeks were colonized with fecal contents harvested from 10 week old male and female C3H/HeNTac ASF mice. Fecal samples were collected at various time points from each individual mouse (h=hour; d=day). qRT-PCR results for ASF 360, ASF 361, ASF 457, and ASF 492 at different time points during colonization are shown for male and female C57BL/6J mice. Data are expressed as means ± SEM.

To transfer ASF from commercially available C3H/HeNTac mice (Taconic) into the C57BL/6J mouse strain, we gavaged GF C57BL/6J mice with the fecal content of the ASF-bearing C3H/HeNTac mouse strain. Remarkably, using species-specific primers for the variable regions V1 through V3 of the bacterial 16S-rDNA [50], we detected the amplicons of all bacterial taxa of the ASF in fecal preps of the F1 C57BL/6J offspring by qRT-PCR, demonstrating the efficient transfer of live ASF bacteria (Figure 1A). While the amplicons of ASF 356 and ASF 500 were weak, ASF 360, ASF 361, ASF 457, ASF 492, ASF 502, and ASF 519 showed more intense bands with the species-specific primers used (Figure 1A). Comparing the ASF-colonized C3H/HeNTac with ASF-colonized C57BL/6J mice, qRT-PCR analysis, and normalization against universal 16S rDNA primers showed that the relative abundance of most bacterial taxa was remarkably constant (Figure 1B). Strikingly, the normalized Ct-value of ASF 500 in the fecal microbiota was elevated in C57BL/6J mice relative to the original C3H/HeNTac donor mouse strain (Figure 1B). By normalizing to universal 16S rDNA primers, we recorded an increase of the individual bacterial taxa with time (Figure 1C). We found a steep increase of ASF 457 at 24 h post gavage, while ASF 360 and ASF 361 steadily increased until day 3 post-gavage. Interestingly, ASF 492 increased between day 3 and day 7 post-gavage but was undetectable before day 3. Hence, our results suggest that the growth of ASF 492 may depend on the quantitative presence of ASF 457, ASF 360, and ASF 361. Using ASF as a minimal microbial consortium with low complexity, our results demonstrate that mouse genetics can determine the abundance of individual gut bacteria.

### 3.2. Altered Schaedler Flora-Colonization Protects from Leukocyte Adhesion to Mesenteric Venules, Prior to and after Ischemia-Reperfusion Injury

**Figure 2:**
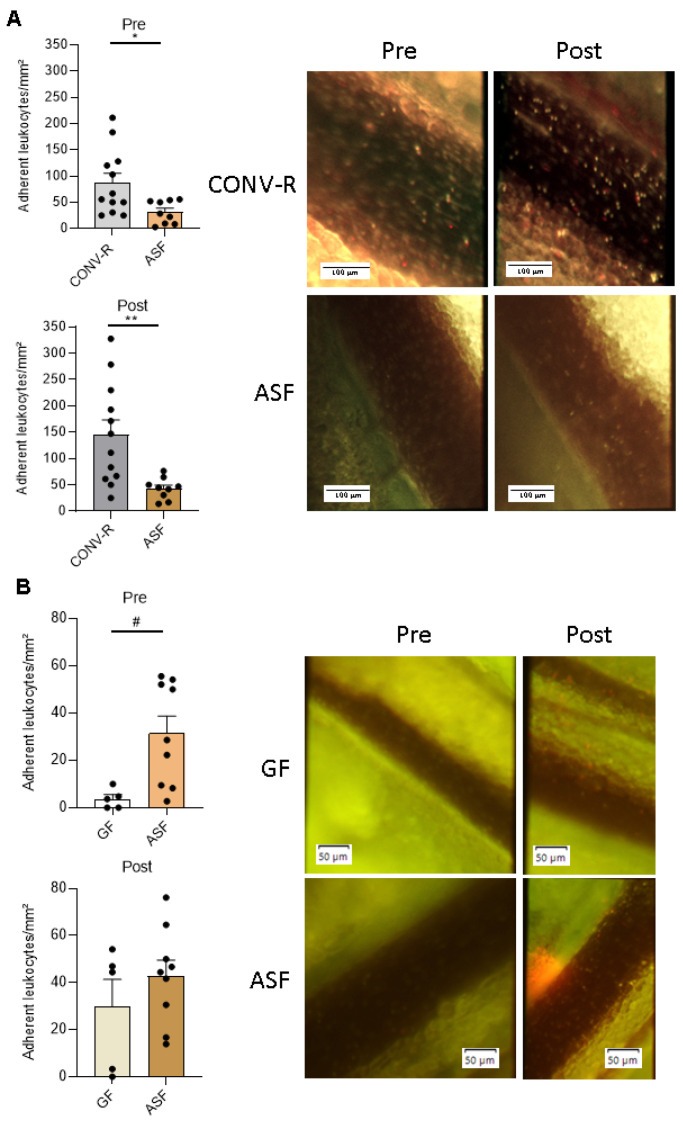
Leukocyte tethering to ischemia-reperfusion-injured mesenteric venules of gnotobiotic ASF-colonized mice. (**A**). Comparative in vivo analysis of adherent leukocytes in the mesenteric venules of CONV-R C57BL/6J (N = 12 mice per group) vs. ASF-colonized C57BL/6J mice (N = 9 mice per group), pre- and post-ischemia-reperfusion injury. Representative images are shown. Adhering leukocytes were stained with acridine orange (green). Scale bar: 100 µm. (**B**). Comparative in vivo analysis in the mesenteric venules of GF C57BL/6J (N **=** 5 mice per group) vs. ASF-colonized C57BL/6J mice (N = 9 mice per group), pre- and post-ischemia-reperfusion injury. Adhering leukocytes were stained with acridine orange (green). Scale bar: 50 µm. All data are expressed as means ± SEM. Statistical comparisons were performed using Student’s *t*-test (* *p* < 0.05, ** *p* < 0.01) or Mann–Whitney test (# *p* < 0.05).

Since our gnotobiotic approach recently demonstrated a pro-adhesive role of a complex microbiota and of *Escherichia coli* JP313 and *Bacillus subtilis* PY79 in leukocyte adhesion in the mesenteric venules [44], we wondered whether the presence of the defined minimal microbial consortium ASF would impact on leukocyte adherence. Using high-speed fluorescence intravital microscopy, we observed a significantly reduced adhesion of leukocytes to the wall of mesenteric venules pre- and post-ischemia in ASF-colonized mice as compared to CONV-R mice that were colonized from birth with a complex gut microbiota (Figure 2A). Pre-ischemia, CONV-R mice showed about two-fold elevated leukocyte counts relative to ASF-colonized mice (*p =* 0.0191). Post-ischemia, adhering leukocytes in the mesenteric venules of CONV-R mice were more than three-fold elevated relative to the ASF-colonized mice (*p =* 0.0057). In contrast to CONV-R mice, ischemia-reperfusion injury only modestly increased leukocyte adhesion in the mesenteric venules of the ASF-colonized mice. Compared to GF housing conditions, the ASF-colonized mice showed significantly increased counts of tethering leukocytes in mesenteric venules prior to ischemia-reperfusion injury (*p =* 0.0185) (Figure 2B). This marked difference was abolished post-ischemia (*p =* 0.5365) (Figure 2B). Taken together, our in vivo results indicate a protective role of ASF colonization in ischemia-reperfusion-induced vascular inflammation.

### 3.3. Altered Schaedler Flora Modulates the Expression of Epithelial-Derived Leukocyte-Attracktant Chemokine CCL5

**Figure 3:**
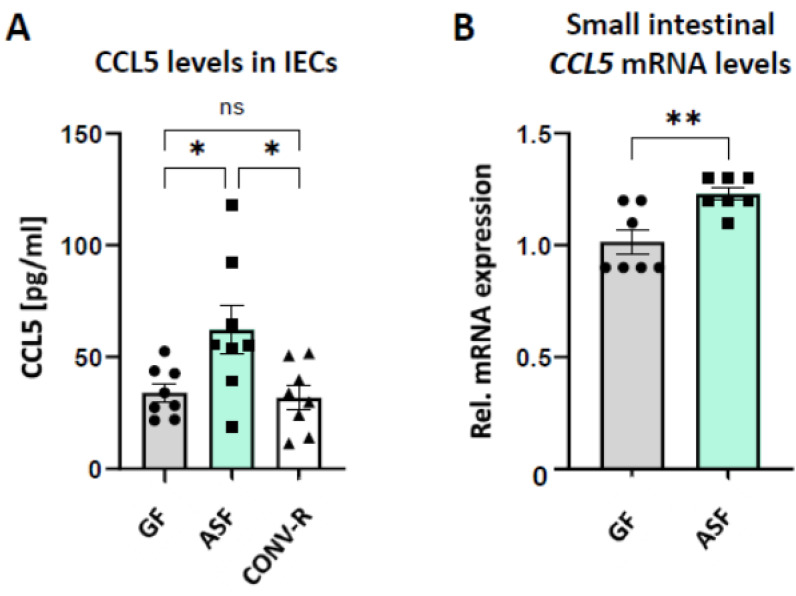
Expression of CCL5 (RANTES) in isolated small intestinal epithelial cells and in the mid small intestine of gnotobiotic ASF-colonized mice relative to GF controls. (**A**). ELISA quantification of CCL5 protein levels in primary intestinal epithelial cell lysates from GF, ASF-colonized, and CONV-R C57BL/6J mice (N = 8 mice per group). (**B**). qRT-PCR quantification of small intestinal *CCL5* mRNA levels in GF relative to ASF-colonized C57BL/6J mice (N = 7 mice per group). All data are expressed as means ± SEM. Statistical comparisons were performed using one-way ANOVA (* *p* < 0.05, ** *p* < 0.01) or Student’s *t*-test (** *p* < 0.01).

C–C motif chemokine ligand 5 (CCL5, RANTES) [51], a gut epithelial-derived chemokine attracting a wide range of leukocytes, was previously demonstrated to promote the interaction of endothelial cells with leukocytes in the small intestinal microvasculature [52]. Therefore, we isolated primary small intestinal epithelial cells from GF, ASF-colonized, and CONV-R C57BL/6J mice and performed ELISA measurements to determine the levels of CCL5. Interestingly, in line with increased leukocyte adhesion in the venules of ASF-colonized mice as compared to GF controls (Figure 2B), CCL5 protein levels were significantly increased in the small intestinal epithelium of ASF-colonized mice, relative to GF and CONV-R mice (Figure 3A). In accordance, small intestinal CCL5 mRNA expression levels were increased in ASF-colonized mice relative to GF controls (Figure 3B), implying that ASF upregulates CCL5 expression levels, thus contributing to the vascular inflammation of mesenteric venules in mesenteric ischemia-reperfusion injury.

## 4. Discussion

Our results demonstrate the successful transmission of ASF from the C3H/HeNTac founder strain to the GF C57BL/6J mouse line at gnotobiotic isolator conditions. Remarkably, the normalized Ct-value of ASF 500 was elevated in the C57BL/6J mouse line relative to C3H/HeNTac mouse strain. Most interestingly, ASF-colonized mice had a reduced leukocyte adherence to ischemia-reperfusion-injured mesenteric venules as compared to CONV-R mice, and ischemia-reperfusion injury resulted in a modest increase in leukocyte adherence in ASF-colonized mice, suggesting a protective role for ASF.

Based on the ASF-colonized C3H/HeNTac mouse strain purchased from Taconic, we successfully colonized C57BL/6J mice by oral gavage of a fecal suspension. PCR analysis with species-specific primers for the V3 through V4 region of the bacterial 16S rDNA proved that all eight taxa were present in the colonized C57BL/6J mouse line. In the time-line sampling qRT-PCR analysis on feces from ASF-colonized C57BL/6J mice, we did not find a sex-specific difference in the time-dependent abundance of individual members of that minimal microbial consortium. ASF 356 (*Clostridium* sp.), ASF 500 (*Firmicutes* sp.), ASF 502 (*Clostridium* sp.), and ASF 519 (*Parabacteroides* sp.) were below the detection limit of this qRT-PCR experiment. Interestingly, in this time-resolved analysis, we observed that ASF 457 (*Mucispirillum schaedleri*) flourished directly at day 1 post gavage, followed by ASF 360 (*Lactobacillus* sp.), and ASF 361 (*Lactobacillus murinus*). ASF 492 (*Eubacterium plexicaudatum)* increased after day 3 post gavage, while ASF 457, ASF 361, and ASF 360 did not increase any further in abundance. This indicates that ASF 492 (*Eubacterium* sp.) may depend on cross-feeding from the other bacterial species, a relevant aspect in ASF that was recently supported by co-culture experiments [53]. In addition to ASF 492, this study identified ASF 519 (*Parabacteroides* sp.) as a butyrate producer. Obviously, host genetics, as evident in the C3H/HeNTac vs. C57BL/6J comparison for ASF 500 (*Pseudoflavinifractor* sp.), is a relevant determinant that can specifically favor the abundance of certain ASF members [50]. As a consequent next step, competition and collaboration within the ASF should be studied in further detail to take advantage of this model system to understand the interdependence of individual ASF members.

In previous work, the minimal microbial consortium ASF turned out to be instrumental for studying how metabolic inflammation, governed by the interplay of diet and specific gut commensals, affects host pathophysiology [54,55]. Invading microbes that enter the circulation trigger thrombotic events in the microcirculation through mechanisms of immunothrombosis [56]. However, so far it remains poorly resolved how gut commensals interfere with inflammatory phenotypes of the vascular endothelium. By high-speed fluorescence intravital microscopy, we could demonstrate here that ASF-colonized mice, in contrast to GF controls, show increased leukocyte deposition to the ischemia-reperfusion injured mesenteric venules (Figure 2B). However, in contrast to CONV-R mice, ASF-colonized mice were protected from leukocyte adhesion in mesenteric ischemia-reperfusion injury. One decisive factor mediating the interaction of leukocytes with the activated endothelium of the gut microvasculature is the chemokine CCL5 [52], which can be synthetized by small intestinal epithelial cells (Figure 3). Interestingly, our analyses demonstrated the ASF-triggered expression of CCL5 in small intestinal epithelial cells, a factor that could augment leukocyte adherence in the small intestinal microcirculation [52,57]. Our results suggest that epithelial CCL5 stimulates microbiota-induced activation of the endothelium of mesenteric venules, thus promoting epithelial–endothelial communication. Enhanced leukocyte tethering could be due to altered surface expression of leukocyte adhesion receptors by the endothelium of mesenteric venules, such as PSGL-1 or ICAM-1 [28,44]. Our previous work identified that tonic microbiota-derived LPS could be critical in tuning leukocyte adherence to the microvascular endothelium [44].

In conclusion, our study demonstrates a protective role of ASF against the observed ischemia-reperfusion-induced increase in leukocyte adherence to the injured endothelium of mesenteric venules. Although we found epithelial-derived CCL5 levels increased in ASF-colonized mice and this chemokine was previously demonstrated to augments leukocyte adherence, its functional involvement and the role of additional microbiota-related factors promoting endothelial cell activation in ischemia-reperfusion-injured mesenteric venules await further investigation. In future studies, our fluorescence microscopy-based in vivo analyses, indicating a protective function for ASF with regard to leukocyte adherence relative to CONV-R housing conditions, should be corroborated by additional methods. In addition, the involvement of this minimal microbial consortium in the regulation of epigenetic mechanisms should be explored [24].

A limitation of our study, as well as of many other studies investigating the impact of microbiota on pathomechanisms, is the fact that clinical data supporting the relevance of our findings are currently missing [43]. A strength of our study is the combination of a well-standardized in vivo mesenteric ischemia-reperfusion model with established gnotobiotic mouse models. Interestingly, our findings are in support of a previous report, demonstrating that depletion of the gut microbiota by antibiotics attenuates intestinal inflammation in ischemia-reperfusion injury [58]. Moreover, our data are in line with the observed protective role of the probiotic formulation VSL#3 in intestinal inflammation-dependent microvascular thrombosis [46]. A further limitation is the sampling of feces, which may interfere with the quantitative detection of ASF community members. Moreover, the SPF CONV-R group, analyzed by high-speed epifluorescence intravital microscopy in the mesenteric ischemia-reperfusion model, lacked an in-depth analysis of microbial diversity. Clearly, future investigations should explore the mechanisms of epithelial–endothelial communication triggered by ASF and delineate their pathophysiological roles in vascular inflammation, employing a tiered experimental pipeline from in silico to in vitro and in vivo. Despite these potential limitations, we feel that our study will stimulate the field to further dissect the protective role of the minimal microbial consortium ASF in mesenteric ischemia-reperfusion injury in the context of various diseases.

## Figures and Tables

**Table 1:** Primers used for the detection of ASF species [50].

Bacterial Species	Taxon ID	Primmer Sequences (5′ to 3′)	Amplicon Size (bp)
*Clostridium* sp.	356	Forward: AAAATAATTAGGAGCTTGCTTGCTTTTAA Reverse: TTAGAAGATGCCTCCTAAGAACC	138
*Lactobacillus intestinalis*	360	Forward:GGTGATGACGCTGGGAAC Reverse: AAGCAATAGCCATGCAGC	130
*Lactobacillus murinus*	361	Forward: GAACGAAACTTCTTTATCACC Reverse: TAGCATAGCCACCTTTTACA	146
*Mucispirillum schaedleri*	457	Forward: TCTCTTCGGGGATGATTAAAC Reverse: AACTTTTCCTATATAAACATGCAC	135
*Eubacterium plexicaudatum*	492	Forward:AATTCCTTCGGGGAGGAAGC Reverse: TAAAACCATGCGGTTTTAAAAAC	137
*Pseudoflavonifractor* sp.	500	Forward:ACGGAGGACCCCTGAAGG Reverse: AGCGATAAATCTTTGATGTCC	172
*Clostridium* sp.	502	Forward:GAGCGAAGCACTTTTTTAGAAC Reverse: TTACACCACCTCAGTTTTTACC	177
*Parabacteroides goldsteinii*	519	Forward:GCAGCACGATGTAGCAATACA Reverse: TTAACAAATATTTCCATGTGGAAC	144

## Data Availability

The data presented in this study are available on request from the corresponding author.
